# The impact of childhood vaccines on bacterial carriage in the nasopharynx: a longitudinal study

**DOI:** 10.1186/s12982-014-0022-3

**Published:** 2015-01-16

**Authors:** Christian Bottomley, Abdoulie Bojang, Peter G Smith, Ousainou Darboe, Martin Antonio, Ebenezer Foster-Nyarko, Beate Kampmann, Brian Greenwood, Umberto D’Alessandro, Anna Roca

**Affiliations:** MRC Tropical Epidemiology Group, Faculty of Epidemiology and Population Health, London School of Hygiene & Tropical Medicine, London, UK; Medical Research Council Unit, Banjul, The Gambia; Faculty of Infectious and Tropical Diseases, London School of Hygiene & Tropical Medicine, London, UK

**Keywords:** Non-specific vaccine effects, Measles, DTP, Nasopharyngeal bacterial carriage

## Abstract

**Background:**

There is increasing evidence that childhood vaccines have effects that extend beyond their target disease. The objective of this study was to assess the effects of routine childhood vaccines on bacterial carriage in the nasopharynx.

**Methods:**

A cohort of children from rural Gambia was recruited at birth and followed up for one year. Nasopharyngeal swabs were taken immediately after birth, every two weeks for the first six months and then every other month. The presence of bacteria in the nasopharynx (*Haemophilus influenzae, Streptococcus pneumoniae, Staphylococcus aureus*) was compared before and after the administration of DTP-Hib-HepB and measles-yellow fever vaccines.

**Results:**

A total of 1,779 nasopharyngeal swabs were collected from 136 children for whom vaccination data were available. The prevalence of bacterial carriage was high: 82.2% *S. pneumoniae,* 30.6%*, S.aureus*, 27.8% *H. influenzae*. Carriage of *H. influenzae* (OR = 0.36; 95% CI: 0.13, 0.99) and *S. pneumoniae* (OR = 0.25; 95% CI: 0.07, 0.90) were significantly reduced after measles-yellow fever vaccination; while DTP-Hib-HepB had no effect on bacterial carriage.

**Conclusions:**

Nasopharyngeal bacterial carriage is unaffected by DTP-Hib-HepB vaccination and reduced after measles-yellow fever vaccination.

## Background

Measles vaccination campaigns conducted in sub-Saharan Africa during the 1980s were associated with substantial reductions (30-50%) in overall childhood mortality. The effect was much larger than was anticipated from protection against measles, and it was suggested that measles vaccine might confer benefits additional to its impact on measles [1]. Observational studies and randomized trials which compared mortality in vaccinated and unvaccinated children have provided further data on the non-specific effects of vaccines in countries where infant mortality is high; diphtheria-tetanus-pertussis (DTP) vaccination was associated with higher childhood mortality [1,2], and the benefits of measles vaccination and adverse effects of DTP were greater in girls than boys [2]. However, it remains unclear how vaccines might contribute to, or prevent, deaths unrelated to the target disease. One possibility is that the vaccines alter susceptibility to bacterial diseases, which are a common cause of child mortality in Africa [3].

Asymptomatic bacterial infections are common in healthy infants and children, especially in developing countries, and a high prevalence of carriage is associated with high burdens of invasive bacterial diseases, which suggests that carriage is a pre-cursor for invasive bacterial disease [4-6]. We are not aware of any previous study that has assessed a possible effect of routine infant vaccination on the risk of bacterial carriage. We have therefore analyzed the association between infant vaccinations and nasopharyngeal carriage of *Streptoccocus pneumoniae*, *Haemophilus influenzae* and *Staphylococcus aureus,* three pathogens commonly associated with invasive disease and mortality in infants and young children in Africa.

## Methods

### Ethics statement

Written parental consent was obtained from a parent or guardian of all study participants. The original carriage study was approved by the joint Medical Research Council (MRC)/Gambia Government Ethics Committee and by the ethics committee of the London School of Hygiene & Tropical Medicine. The retrospective collection of vaccination dates was approved by the joint Medical Research Council (MRC)/Gambia Government Ethics Committee.

### Participants and study design

Between December 17^th^, 2003, and June 16^th^, 2005, newborn infants were recruited from 21 villages in the Sibanor region, The Gambia in preparation for a cluster randomized trial of a seven-valent pneumococcal conjugate vaccine [7,8]. Nasopharyngeal swabs (NPS) were taken as soon as possible after birth, every two weeks for the first six months, and then every other month until the first birthday.

As part of the Expanded Programme on Immunisation (EPI), children were scheduled to receive BCG at birth or soon afterwards, followed by three doses of a combined DTP, *H. influenzae* type b (Hib) and hepatitis B vaccine (starting at two months and then monthly). Measles and yellow fever vaccines were scheduled at the age of 9 months. Poliomyelitis vaccine was administered at birth, and then at two, three, four, 9 and 12 months of age. The dates of all vaccinations, with the exception of those for polio, were retrospectively transcribed in 2012 by study field assistants from either the welfare card of the participants or health centre records.

### Sample collection

Nasopharyngeal swabs (NPSs) were collected from the posterior wall of the nasopharynx using a calcium alginate swab, and immediately inoculated into vials containing skim-milk-tryptone-glucose-glycerol (STGG) transport medium; these were then placed in a cold box before being transferred to the MRC Unit Fajara laboratories within eight hours of collection. The procedure was conducted in accordance with the World Health Organization protocol for evaluation of pneumococcal carriage [9]. Inoculated vials were vortexed for a minimum of 20 seconds to homogenize the bacterial suspension before being stored at −70°C until they were tested in batches.

### Laboratory procedures

The samples were analyzed for the presence of *S. pneumoniae* as part of the original study by Hill *et al.* [7]. In 2012, the STGG samples were thawed on ice, vortexed briefly, and inoculated onto agar plates for the isolation of *S. aureus, H. influenzae* and β-haemolytic streptococci.

*Streptococcus pneumoniae.* Ten μl of a thawed STGG sample were plated onto gentamicin blood agar and incubated for 18-24 hours at 35°C in 5% CO_2_. Pneumococcal identification was based on colony morphology, and conventional methods of characterization (optochin susceptibility and bile solubility assays) [10]. Serotyping was performed with capsular and factor typing sera (Statens Serum Institut) using the latex agglutination technique [10]. Isolates with equivocal serotype results were confirmed by the Quellung reaction.

*Staphylococcus aureus.* Fifty μl of thawed STGG sample were plated onto mannitol salt agar plates and incubated for 48 hours at 37°C in ambient air. The plates were examined for yellow or white colonies typical of staphylococci, and subcultured onto blood agar plates to obtain pure growth. A catalase test was performed on all suspected colonies, followed by coagulase testing using the Remel Staphaurex® Plus kit (Oxoid cat# OXR30950201), to confirm *S. aureus*.

*Haemophilus influenzae.* Fifty μl of thawed STGG sample were plated onto bacitracin supplemented chocolate agar plates and incubated at 37°C with 5% CO_2_. After 18-24 hours, the plates were examined for colourless or grey, translucent or opaque colonies, and these were sub-cultured onto chocolate agar. An oxidase test was performed on all presumptive *H. influenzae* colonies. Oxidase positive colonies were screened using X and V factors to confirm the isolation of *H.influenzae*. Confirmed isolates were further serotyped using BD Difco^TM^ Hi-antisera Type a-f.

*Beta-haemolytic streptococci.* Fifty μl of thawed STGG sample were plated onto crystal violet supplemented blood agar for 20-24 hours and incubated at 37°C in ambient air. Plates were examined for colonies showing transparent zones around them (beta-haemolysis); these were sub-cultured onto blood agar plates to obtain pure growth. Beta-haemolytic and catalase negative isolates were subjected to Bacitracin testing and classified using the slidex streptococcal grouping kit (Biomerieux, cat# 58810) into Lancefield groups A, B, C, D, F or G.

### Statistical analysis

We analysed the prevalence of bacterial carriage in each decile of age – i.e., the ages at which samples were taken were split into 10 groups of equal size with the first group containing the 10% of samples taken at the youngest ages, the second group containing those taken at the next youngest 10%, and so on. Confidence intervals for these prevalence estimates were adjusted for clustering at the village level.

In addition, we used logistic regression models, in which age was included as a restricted cubic spline function, to estimate age-prevalence curves. Restricted cubic splines are used to model the relationship between an outcome (carriage) and a continuous predictor (age). Specifically, the restricted cubic spline splits the continuous predictor into categories and uses separate cubic relationships to model the association within each category, except in the first and last category where a linear relationship is assumed [11].

To estimate the effect of DTP (first dose and combined with Hib and hepatitis B) vaccination on carriage, we compared the prevalence of bacterial carriage before and after DTP vaccination. We excluded samples collected before BCG or after measles-yellow fever vaccination, and samples collected after the first DTP-Hib-HepB vaccination were divided into those collected <4 weeks or ≥4 weeks after vaccination to determine whether any effect of DTP-Hib-HepB changed with time since vaccination. Confounding attributable to variability between individuals was eliminated by estimating the effect of vaccination on bacterial carriage within individuals using conditional logistic regression. Samples before DTP-Hib-HepB vaccination were necessarily taken at a younger age than those after DTP-Hib-HepB vaccination and to adjust for the potential confounding effect of age we included age in the model as a restricted cubic spline function with six knots (i.e., six cut points), as recommended by Harrell [12], located at percentiles 5, 23, 41, 59, 95. Potential confounding due to seasonality was accounted for by including calendar time in the model as a restricted cubic spline function with three knots at percentiles 10, 50 and 90.

The effect of measles-yellow fever vaccination was estimated by comparing samples collected after BCG + DTP-Hib-HepB with those obtained after BCG + DTP-Hib-HepB + measles-yellow fever vaccination. Conditional logistic regression models were used to estimate odds ratios as described above for DTP-Hib-HepB.

All analyses were conducted using Stata version 12.1 (StataCorp).

## Results

### Demographic data

There were 251 live births in the study population during the 18-month recruitment period, 237 (94.4%) infants were recruited into the study, and at least one sample was collected from 236 infants. The analyses reported here are based on the cohort of 136 (57.6%) infants for whom all vaccination dates were recorded; children who missed one or more vaccinations, or who did not have a complete record of vaccinations based on their welfare card and clinic records, were excluded. Slightly less than half of the cohort (46.3%) was female and the predominant ethnicities were Jola (67.6%) and Madinka (22.1%). The median (IQR) birth weight, recorded by the study team at the mother’s home or in the health facility, was 3.9kg (3.4, 4.4), and 3.7% of infants were of low birthweight (<2.5kg).

### Age at vaccination

**Figure 1:**
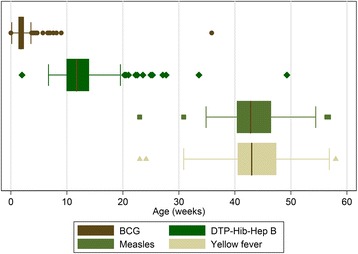
**Box and whisker plot for ages at vaccination (first vaccine dose in the case of DTP-Hib-Hep B).** The “box” represents the interquartile range (IQR) and the whiskers span all points within 1.5 IQR.

There was variability in the ages at which each of the vaccines was administered (Figure 1). The median (IQR) age of vaccination was 1.8 weeks (1.4, 2.3) for BCG, 11.7 weeks (10.0, 13.9) for DTP-Hib-Hep B (first vaccination), 42.8 weeks (40.4, 46.4) for measles and 43.0 (40.6, 47.4) for yellow fever. Yellow fever vaccine was generally given concurrently with measles, but was occasionally given later if it was not available at the clinic at the time of measles vaccination. Because the age range over which BCG was given was small, we have not included analyses of carriage data before and after BCG vaccination.

### Bacterial carriage and its age-profile

A total of 1,779 samples were collected. The number collected from each child varied from 2 to 17, with a median of 14 (IQR: 11, 15) samples per child. The proportions of samples positive for *S. pneumoniae*, *H. influenzae*, *S. aureus* and Group B streptococcus (GBS) were 82.2%, 27.8%, 30.6% and 1.6%, respectively. GBS was excluded from further analyses because of its low prevalence. Among the *S. pneumoniae* positive samples, the most common serotypes were 6B (15.0%), 19F (11.4%), 14 (9.3%), 6A/C (7.6%) and 19A (5.1%). Thirty-seven isolates of *H. influenzae* were serotyped: 2 (5.4%) were serotype E, 1 (2.7%) serotype F and the rest (n = 34) were non-typeable.

**Figure 2:**
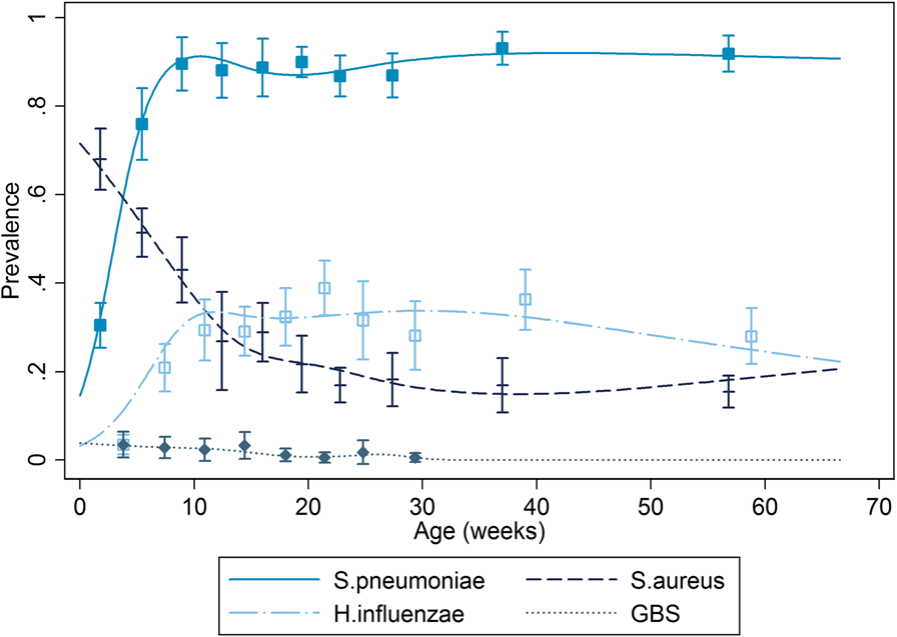
**The prevalence of bacterial carriage during follow up.** The prevalence is given in each decile of age with 95% confidence intervals, the lines are predictions from a logistic regression where the effect of age on carriage is modeled using spline functions.

The prevalence of *S. aureus* declined from approximately 70% immediately after birth to 20% at 20 weeks and then remained constant until the end of follow up at 1 year (Figure 2). In contrast, the prevalences of *S. pneumoniae* and *H. influenzae* increased after birth, to reach a plateau between 10 and 20 weeks of approximately 85% and 35%, respectively.

### Vaccination and bacterial carriage

Table 1 shows the prevalence of carriage before and after the first dose of DTP-Hib-Hep B vaccination and the odds ratios comparing prevalence post and pre vaccination after adjusting for age and calendar time. There was no evidence of a significant change in the prevalence of carriage for any of the three bacteria. Table 2 shows similar data for measles-yellow fever vaccination. Carriage of *S. pneumoniae* and *H. influenza*e decreased after measles-yellow fever vaccination. For *H. influenza*e the greatest reduction occurred in the first month after vaccination, while for *S. pneumonia* the reduction was similar regardless of when samples were collected. Measles-yellow fever vaccination had no effect on the prevalence of *S. aureus*. The analyses were repeated separately for boys and girls, but there was no significant difference between the findings for each gender (data not shown). We also conducted analyses where age was modeled as a categorical variable using deciles, the results of these analyses were similar to those presented in Tables 1 and 2 where age was modeled using spline functions.

**Table 1:** **Bacterial carriage before and after the first dose of DTP-Hib-Hep B vaccination**

**Period**	**No. samples**	**Prevalence (%)**	OR* (95% CI)	**p-value**
*S. pneumoniae*				
Pre vaccination†	518	86.6		
Post vaccination (<4wks)	201	87.6	0.99(0.39,2.49)	0.975
Post vaccination (4 + wks)	767	90.4	1.83(0.65,5.15)	0.252
Post vaccination (All)	968	89.8	1.36(0.65,2.86)	0.411
*H. influenzae*				
Pre vaccination†	512	33.3		
Post vaccination (<4wks)	199	30.2	1.03(0.57,1.86)	0.923
Post vaccination (4 + wks)	753	34.4	1.48(0.74,2.97)	0.270
Post vaccination (All)	952	33.5	1.02(0.62,1.69)	0.928
S. *aureus*				
Pre vaccination†	512	28.4		
Post vaccination (< 4wks)	199	29.6	0.97(0.54,1.74)	0.924
Post vaccination (4 + wks)	753	19.0	0.86(0.41,1.79)	0.680
Post vaccination (All)	952	21.2	0.97(0.57,1.63)	0.897

Based on an analysis of 123 individuals. Samples collected post BCG compared with samples collected after the first DTP-Hib-Hep B vaccination and prior to measles-yellow fever vaccination.

*OR adjusted for age and calendar time. Estimates from each model are based on different numbers of individuals because information is not contributed by individuals whose carriage status is the same at all time points; estimates for *S.pneumoniae*, *H. influenzae* and *S.aureus* are based on 1062, 1388, 1398 samples from 93, 125, 124 individuals.

† prevalence in samples collected after 10 weeks based on 142, 141 and 141 samples for *S.pneumoniae*, *H. influenzae* and *S.aureus.*

**Table 2:** **Bacterial carriage before and after measles-yellow fever vaccination**

**Period**	**No. samples**	**Prevalence (%)**	OR* (95% CI)	**p-value**
*S. pneumoniae*				
Pre vaccination†	968	93.8		
Post vaccination (< 4wks)	48	83.3	0.19(0.05,0.72)	0.014
Post vaccination (4 + wks)	107	91.6	0.19(0.03,1.35)	0.098
Post vaccination (All)	155	89.0	0.25(0.07,0.90)	0.033
*H. influenzae*				
Pre vaccination^†^	952	41.1		
Post vaccination (< 4 wks)	47	14.9	0.36(0.13,0.99)	0.047
Post vaccination (4 + wks)	106	29.2	0.80(0.32,1.97)	0.625
Post vaccination (All)	153	24.8	0.53(0.25,1.12)	0.097
*S. aureus*				
Pre vaccination†	952	18.9		
Post vaccination (<4 wks)	46	15.2	1.15(0.36,3.70)	0.816
Post vaccination (4 + wks)	106	17.0	1.04(0.32,3.31)	0.951
Post vaccination (All)	152	16.4	1.05(0.41,2.73)	0.916

Samples collected after vaccination with DTP-Hib-Hep B compared with samples post measles-yellow fever.

*OR adjusted for age and calendar time. Estimates from each model are based on different numbers of individuals because information is not contributed by individuals whose carriage status is the same at all time points; estimates for *S.pneumoniae*, *H. influenzae* and *S.aureus* are based on 641, 1039, 922 samples from 70, 119, 104 individuals*.*

^†^prevalence after 35 weeks based on 97, 95 and 95 samples for *S.pneumoniae*, *H. influenzae* and *S.aureus*.

## Discussion

To our knowledge, this is the first report that measles-yellow fever vaccination may reduce the bacterial carriage of some pathogens. The nasopharynx acts as a reservoir for a number of pathogenic bacteria and asymptomatic nasopharyngeal carriage, even if transitory, is a necessary preliminary step in the progression to severe disease. The relationship between bacterial carriage and disease is illustrated by a parallel decrease in carriage of vaccine serotypes and invasive pneumococcal disease following the introduction of pneumococcal conjugate vaccines [6]. Changes in the prevalence of bacterial carriage are, therefore, likely to affect the incidence of severe disease and mortality, although our study was too small to assess these outcomes.

Our findings support a possible protective effect of measles-yellow fever vaccination against disease due to *S. pneumoniae* and *H. influenzae*, which would be compatible with a non-specific effect of measles vaccination on diseases other than measles. In contrast, our failure to find an association between DTP-Hib-HepB vaccination and carriage of any of the three bacteria studied offers no support to the hypothesis of a deleterious non-specific effect of DTP vaccination [13]. It should be noted that because measles vaccination was generally given with yellow fever vaccination we cannot distinguish between effects of the two vaccines, similarly DTP vaccination was given with Hib and Hepatitis B vaccination so any individual effects of these three vaccines also cannot be distinguished.

The mechanisms underlying the observed non-specific effect of measles-yellow fever vaccination on *S.pneumoniae* and *H.influenzae* carriage are unknown. The immune response to measles vaccine is characterized by generalized immunosuppression, but also a strong Th-1 cytokine pattern [14], which might impact on carriage. The live attenuated yellow fever vaccine, YF-17D, activates the innate immune system and induces a mixed T helper 1 and T helper 2 cell profile [15]. Furthermore, an interaction between YF-17D and dengue has been documented [16].

The vaccine effect was determined by comparing carriage status before and after vaccination. This approach is different to that taken in previous observational studies which have compared vaccinated and unvaccinated children; such estimates can be biased by differences in the socio-economic or underlying health status of the two groups [17]. By comparing samples collected within the same children we eliminate bias of this kind, although the estimates are still vulnerable to time-dependent confounders such as age, season, delayed vaccination or use of antibiotics [18]. Seasonality has a limited role as a confounder since births and vaccinations occurred at a uniform rate throughout the study. Age is potentially an important confounder but the prevalence of *H. influenzae* and *S. pneumoniae* remained approximately constant after10 weeks, so this is unlikely to explain the observed reduction after measles-yellow fever vaccine; furthermore we were able to control for the effect of age using regression models. It is unclear to what extent delayed vaccination and the use of antibiotics might confound our estimates. Carriage increases during a respiratory infection and if vaccination is delayed when a child is sick then this might explain the decrease in bacterial carriage [18]; alternatively, if a sick child is given antibiotics at the time of vaccination then this will also lead to a decrease in bacterial carriage.

A limitation of our study design is that the vaccine effects could not be evaluated separately. We are only able to report the effect of DTP-Hib-HepB in children who have been vaccinated with BCG, and the effect of measles-yellow fever vaccine in children previously vaccinated with BCG and DTP-Hib-HepB. In addition, our analysis cannot differentiate whether the effect observed is due to vaccines that are concurrently given - measles is given with yellow fever and polio is administered with BCG, DTP and measles.

We excluded from the analysis individuals for whom dates of vaccination were missing, either because they did not receive the vaccination, or because the information was missing from the welfare card or clinic records. It is possible that children excluded from the analysis are systematically different from those included (e.g., they might have poorer and less educated mothers), and the association between vaccination and bacterial carriage might be different in those excluded.

## Conclusions

In conclusion, our study provides preliminary evidence for a non-specific effect of measles and/or yellow fever vaccines on bacterial carriage of important bacterial pathogens, which warrants further investigation. Randomized clinical trials would be the best way to test our findings, for example, by examining the effects on carriage of different vaccination schedules.
